# A Qualitative Exploration of Collective Collapse in a Norwegian Qualifying Premier League Soccer Match—The Successful Team's Perspective

**DOI:** 10.3389/fpsyg.2021.777597

**Published:** 2022-01-18

**Authors:** Gaute S. Schei, Tommy Haugen, Gareth Jones, Stig Arve Sæther, Rune Høigaard

**Affiliations:** ^1^Department of Sports Science and Physical Education, University of Agder, Kristiansand, Norway; ^2^School of Sport and Exercise Science, University of Worcester, Worcester, United Kingdom; ^3^Department of Sociology and Political Science, Norwegian University of Science and Technology (NTNU), Trondheim, Norway

**Keywords:** elite sport, soccer, negative momentum, positive momentum, emotional contagion, performance contagion

## Abstract

The current case study focused on a crucial match in the qualification for the Norwegian Premier League (Eliteserien). In the match, the participants of the study experienced a radical change in performance toward the end of the second half, from being behind by several goals to scoring 3 goals in 6 min and winning the qualifying game. The purpose of this study was therefore to examine the perceptions and reflections of players and coaches (sporting director) on what occurred within their own team and within the opposing team. The momentum shift in the opposition team can be described as a collective collapse. In the study, the theoretical collective collapse process model was used as a guide for the design of the interview questions where five semi-structured interviews were conducted with participants involved in the match (players, coach, and sporting director). The participants watched excerpt clips from the match to recall the main events, which they subsequently reflected on. The results highlighted the importance of the “before-game” aspects (i.e., pressure, first game result), the “during-the-game” behavior (i.e., goals scored, playing with a low degree of risk) and the cognitive (i.e., feelings of pressure, despair) and emotional reactions (i.e., frustration, joy) to the match unfolding. In addition, social contagion processes were evident in both teams relating to emotion and behavior. Overall, the data from this study investigated the general structure of the process model of collective sport team collapse and found support for the notion of a temporal cascade of causes for a team collapse. Future research is encouraged to examine this model, to provide guidance to teams, coaches, and sport psychologists in order to make recommendations for dealing with collective collapse in sport teams.

## Introduction

**Figure 1:**
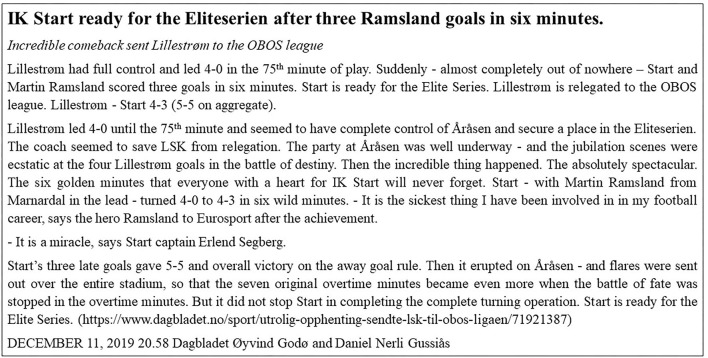
Report in the daily newspaper (translation conducted by the authors). Published with permission from Dagbladet.

What happened in the soccer match between Lillestrøm (LSK) and idrettsklubben (IK) Start (Figure 1) is something that occasionally happens in sport, a sudden and unexpected change in performance. This may be especially pertinent in soccer where the outcome of the match has large financial implications (Hoffmann et al., 2002). The term “momentum” is frequently used when the sequence of scoring or winning (success) has an influence on future performance success (Vallerand et al., 1988; Taylor and Demick, 1994; Gernigon et al., 2010; Briki et al., 2013; Iso-Ahola and Dotson, 2014). According to Higham et al. (2005), a positive momentum can, to some degree, be caused by a negative momentum in the opposition team. An extreme decline in performance and underperformance of many of the players in a team is referred to as collective collapse (Apitzsch, 2006, 2019). Usually, a collective collapse seems to be triggered by a critical situation that disrupts the interaction of the team, wherein they lose control of the match and are unable to regain it. Collective collapses most often occur in matches that are crucial. Particularly, where much is at stake and often with limited opportunity to return to previous levels of performance and is, thus, a more chronic condition (Adler and Adler, 1978; Cotterill, 2012; Den Hartigh et al., 2014).

Wergin et al. (2018) developed a collective collapse process model that distinguishes between precursors (predisposing conditions) and factors that create vulnerability to experiencing collective collapse, critical triggers, and cognitive, emotional, and behavioral outcomes in practitioners. Although the factors in the model are presented in a series of causes, the factors will be linked in dynamic and cyclical processes. Particularly, they influence and reinforce each other. Precursor related factors for a collective collapse that has been reported in the literature are a lack of attentional focus, over confidence, and poor preparation (Apitzsch, 2006; Wergin et al., 2018, 2019). Furthermore, it appears to occur more often in important matches, where a lot is at stake and where there is a lot of pressure. In addition, collective collapse seems to be more prevalent at the end of a match when the players begin to experience physical fatigue (Wergin et al., 2018).

Critical events or triggers are identified as crucial factors that accelerate the process toward a collective collapse (Apitzsch, 2009; Wergin et al., 2018, 2019). Triggers can be when the opposition scores a goal, changes in the tactics, or a key refereeing decision is made. Critical events can also be caused by internal factors. For example, when key players start to make mistakes or that several players make mistakes at the same time (Taylor and Demick, 1994; Jones and Harwood, 2008). In the models by Wergin et al. (2018, 2019), critical incidents can affect the players cognitively, emotionally, and behaviorally. Cognitive outcomes of a critical event may, for example, increase insecurity, lack of accountability, despair, individualization, and pressure that may hinder them to revert to normal performance (Wergin et al., 2018, 2019). For example, increased insecurity and lack of accountability may reduce the responsibility of players to take action and might also be associated with social loafing (Karau and Wilhau, 2020). Emotional outcomes can be frustration and anxiety (Wergin et al., 2019). Several studies have shown that negative emotions are associated with decreased performance (Barsade and Gibson, 2012; Hill and Shaw, 2013; McEwan and Beauchamp, 2014). More specifically, negative emotions contribute to increased uncertainty, confusion, or even panic, and such refocusing and changing to a new situation can be very difficult (Higham et al., 2005; Wergin et al., 2018). The behavioral outcomes of critical incidents can cause hectic play or cautious play (Wergin et al., 2019). Hectic play, for example, can be related to choking. Baumeister and Showers (1986), p. 262 defined choking as “the occurrence of sub-optimal performance under pressure conditions.” When athletes choke under pressure, they begin to focus on their own achievements and become too self-conscious, which can be detrimental to the flow of movement and performance. However, choking may both be an antecedent to collective collapse or a consequence of the collective collapse (Hill et al., 2009; Wergin et al., 2019). Another behavioral outcome of a critical event is that constructive intra-team communication may be less frequent and replaced with negative communication (e.g., blaming, criticism) or silence among the players. Consequently, a negative atmosphere, reduced unity, and a mindset of fear of failure may occur (Apitzsch, 2009; Wergin et al., 2019).

According to research (Apitzsch, 2006; Wergin et al., 2019), a main factor in a collective collapse is the deterioration of the playing structure in the team. Salas et al. (2008) and Filho et al. (2015) stated that shared mental models are required for optimal team performance. Former research has indicated that a lack of operative and appropriate shared mental models is related to role ambiguity, poor interaction and coordination, inappropriate decision making, and reduced team performance (Reimer et al., 2006; Gershgoren et al., 2013; Giske et al., 2017). In team sport, key performance indicators (KPI) are often used to evaluate performance (Menmert and Rein, 2018). Wright et al. (2014) define KPI as factors that are aligned with success for a specific team and individual. To better understand the performance, coaches and performance analysts at the elite level often have large data sets at their disposal. This data focuses predominately on technical, tactical, and physical performance variables (Sarmento et al., 2014). Although these performance variables give an important insight into how the team and the individual has performed, the data does not explain how social and psychological aspects (e.g., emotions and communication) may have affected the flow, momentum, and performance of teams (Wright et al., 2014; Pettersen et al., 2021).

The emotions and behaviors of individual players can, through social contagion, permeate through the whole team. Social contagion has been defined by Levy and Nail (1993), p. 271 as a process where “the spread of affect, attitude, or behavior from Person A (the “initiator”) to Person B (the “recipient”), where the recipient does not perceive an intentional influence attempt on the part of the initiator.” In sport, it has been documented that the contagion effect of individual emotions can affect the emotion of the team (Moll et al., 2010) and subsequently affect team performance (Boss and Kleinert, 2015). For example, Barsade (2002) found that the transfer of positive emotions increases the likelihood of improved cooperation, decreased conflict, and increased perceived task performance. The transfer of negative emotions is labeled as negative emotional contagion (Totterdell, 2000). Of great interest, negative emotions have been reported to spread faster than positive emotions (Tickle-Degnen and Puccinelli, 1999; Baumeister et al., 2001) and negatively influence the cognition of an athlete and promote a downward spiral leading to, or maintaining, a collective collapse (Wergin et al., 2019). In addition to the internal emotional contagion, there may also be an emotional transference between the teams, but then, with a contrasting effect (Taylor and Demick, 1994). For example, in a study by Moll et al. (2010) investigating emotional contagion in penalty shootouts, it was found that there was an expression of positive emotions within one team and increased feelings of inferiority in the opposing team. This indicates that when players demonstrate behaviors of dominance, opposition players may feel less confident, with the probability of success diminishing due to this inter-team contagion process.

Finally, there is also a behavioral contagion in a team where, for example, poor performance can be transferred among the players. When key players make mistakes, the negative transference seems to be more devastating because some players adapt easier to the mood and performance of key players compared to others (Apitzsch, 2009; Wergin et al., 2019). Even if there is a growing knowledge about the collective collapse phenomenon, more empirical research has been requested (Apitzsch, 2019; Wergin et al., 2019). Moreover, most of the research on collective collapse has focused the investigation from the collapsing team perspective and research to investigating how the interactions between the collapsing team and the opposing team unfold prior and during the collapse would be of great interest. Therefore, the purpose of this study was to investigate the perceptions and reflections of players and coach on collective collapse from a bystander perspective.

## Method

### Pre-match Context

December 1, 2019, when the Norwegian football leagues (Eliteserien and first division) played their final rounds, it became clear that LSK from Eliteserien and IK Start from the first division would meet in a two-match qualification series to decide which team would play in Eliteserien in 2020. IK Start finished the first division league in third place and won the qualification battle in the first division between teams 3 and 6, thereby earning the opportunity to participate in the last qualification leg against a team from the Eliteserien. LSK finished their season in 14th place of 16th teams in total after securing one point in the last round of the league, avoiding relegation on goal difference. In the first qualification game between IK Start and LSK, IK Start won their home game 2-1 after being down 0-1 at half time.

### Research Design

Considering the sparse amount of research on the phenomenon of collective collapse in sport, the current study was deemed suitable for a qualitative method of exploration. A phenomenological approach (Kvale and Brinkmann, 2009) was considered the most appropriate for the present study to investigate the reality of participants through their experiences, reflections, and opinions. To be able to explain the investigated phenomenon, an ideographic approach was chosen (Robson and McCartan, 2016). A case study design was used since it is a useful strategy to understand a phenomenon in depth (Yin, 2014). Furthermore, semi-structured interviews were applied to obtain descriptions and interpretations of the phenomena of a collective collapse through the eyes of the interviewees (Kvale and Brinkmann, 2009). With an exploratory research design, data sampling was conducted on three different levels: (1) video recording of the investigated game, (2) objective match statistics of the investigated game conducted by Wyscout, and (3) verbalization during post performance interviews. In addition, expert group discussions were utilized to conduct subjective match-analysis of the examined game to optimize the understanding of researchers on how the game unfolded before the interviews with the participants were undertaken.

### Participants

A purposive sampling approach was guided by the goal of recruiting participants who were particularly knowledgeable about the investigated game and had a central role in the team to maximize the content and the quality of the data (Robinson, 2014). The sample comprised of three players, the head coach, and the sporting director. All participants were male and from the club IK Start. The ages of the players were 22, 26, and 32 years, respectively. They had played for the team on average 2.3 years. The head coach and the sporting director had been in their positions for 1 year, were both former elite soccer players who played for national and international clubs, and have represented their national teams at senior level.

### Procedure

Following ethical approval from the university of the first author and the Norwegian Social Sciences Data Service, the sporting director of IK Start was contacted and permission was given to contact players and coaches in order to arrange interviews. Potential participants were approached (including the sporting director) and informed of the study aim and objectives. All five participants were approached and agreed to contribute before interview arrangements were subsequently sorted. Interviews were conducted during 2 days in February of 2020 during the pre-season, 2 months after “the game” (the subject of the interview) was played. Participants were individually interviewed by two separate researchers in a meeting room in the home stadium of the participants. Informed consents were provided and signed before the interviews commenced. Participants were informed that they could access the transcribed material after the interview to make necessary changes and that they had the freedom to withdraw from the study at any given time. Interviews lasted an average of 48.2 min (range 42–55 min) and were digitally recorded.

### Interview Guide

In relation to developing the interview guide, the process model of causes of collective collapse by Wergin et al. (2018) was examined in addition to former research on collective collapse in sport. This enhanced the understanding of the researcher of the phenomenon of collective collapse for the development of questions and probes for the interview guide prior to the actual interviews.

The interview guide was divided into four different sections. Section 1 included information about the current study, informed participants of their freedom to read through the transcribed material after the interview, and provided opportunity for participants to ask any questions before the interview started. It was clearly specified by the researchers in this section that it is possible to identify the participants in the produced paper. Section 2 consisted of an open conversation about the game from the perspective of the participant and their experience of it (e.g., “What were your thoughts and emotions before the game?”). Questions about different phases and aspects of the game were asked, and the respondents were allowed to talk freely about the key situations as they experienced them during the game. Section 3 involved a systematic video-recall review of the investigated game where the participants were exposed to 11 short clips of game situations that could trigger reflections that had not been mentioned (e.g., what occurred in your team during and after this situation? What did you observe among the opposition when this happened?”). Using video-recordings as stimuli to recall situations has been demonstrated to be a useful technique in semi-structured interviews (Schei and Giske, 2020). In Sections 2 and 3, it was important that the respondent expressed his experience from the following viewpoints: from the perspective of his own team, how they perceived the opposing team, and how both teams reacted to the different situations during the game. Different follow-up questions allowed for further clarification and exploration of key points made in addition to providing important nuances (Patton, 2002). Section 4 included a short summary and final questions (e.g., What was the main reason you managed to turn the game and qualify for the Eliteserien?”). Participants were also given the opportunity to add any other important information that they felt was missing.

### Data Collection

Cognition and experience during the match were examined with the aid of a video montage. The match was video recorded on national television and the film was used to create a video montage. The purpose of the montage was to assist the recall of cognitive information stored in the memory by viewing video footage of the situations under examination. This method has been used effectively in previous research (Trudel et al., 2001). To create the video montage, key critical situations during the match were identified (e.g., goals, substitutions). Video segments were, on average, 22 s long. For example, the clip of a goal also included actions prior to the goal, the shot, and the celebration. The following sections were included in the video montage: (1) before the match, (2) first goal to LSK, (3) second goal to LSK, (4) start of the second half, (5) third goal to LSK, (6) fourth goal to LSK, (7) double substitution for IK Start, (8) first goal to IK Start, (9) second goal to IK Start, (10) third goal to IK Start, and (11) end of the match. The video segments were presented to the participants in their sequence of occurrence during match, and after each segment, the players were challenged to recall and reflect (think out loud) freely about themselves, their own team, and the opposition.

### Data Analysis

The objective of utilizing qualitative analysis was to examine the information embedded in the responses of the athletes to the video montage, which represented their thoughts during the targeted match and the selected critical moments. Transcripts from the interviews were analyzed using thematic analysis, following the six-phase model described by Braun et al. (2016). The analytic process of thematic analysis was deemed useful for identifying common patterns in the current data set. During each interview session, the interviewer made notes on the general points that were being made by the participant. These notes, while not coded, were used to focus the analytic thoughts of the researcher before the formal data analyses occurred. The researchers reviewed the transcripts for a minimum of two times to allow familiarity with the data. Responses to each specific segment of the montage were transcribed verbatim and analyzed independently. The research team met several times to establish a consistent understanding of the data, following phases 1-2 of the thematic analysis (Braun et al., 2016). Once the researchers agreed, raw data was tagged with a descriptive label to represent the type of cognition conveyed by the participants into lower order themes or subthemes. These lower order themes or subthemes were then organized into higher order themes, following phases 3–5 of theme development, refinement, and naming (Nowell et al., 2017). Lastly, the sixth phase of writing up the analysis was completed by connecting results from the data set to the existing literature and compiling the specific different aspects of the current paper (Braun and Clarke, 2006).

Several checks were incorporated to ensure the trustworthiness of the qualitative data interpretation through an interrelated process during different phases of the thematic analysis, following recommendations from Braun et al. (2016) and Nowell et al. (2017). First, prolonged engagement with the data set and triangulation of the collected data was an ongoing process throughout the development of the current research study. During expert group discussion, interview data was interconnected with match recordings and objective match statistics. This process gave an enhanced meaning to the transcribed material by linking data from different perspectives and, therefore, increasing the understanding of the game events. Secondly, peer debriefing and reflexive writing created a foundation for reflection during the process of coding, which improved the structure of the continuing thought process and collation of ideas arising from the raw data (Cutcliffe and McKenna, 1999). Finally, quotes were selected to capture the essence of the developed themes and to provide transparency to the conclusions made by the researchers in the study. Potential researcher disagreements regarding coding, theme development, and conclusions were solved through discussion until consensus was achieved.

## Results

**Figure 2:**
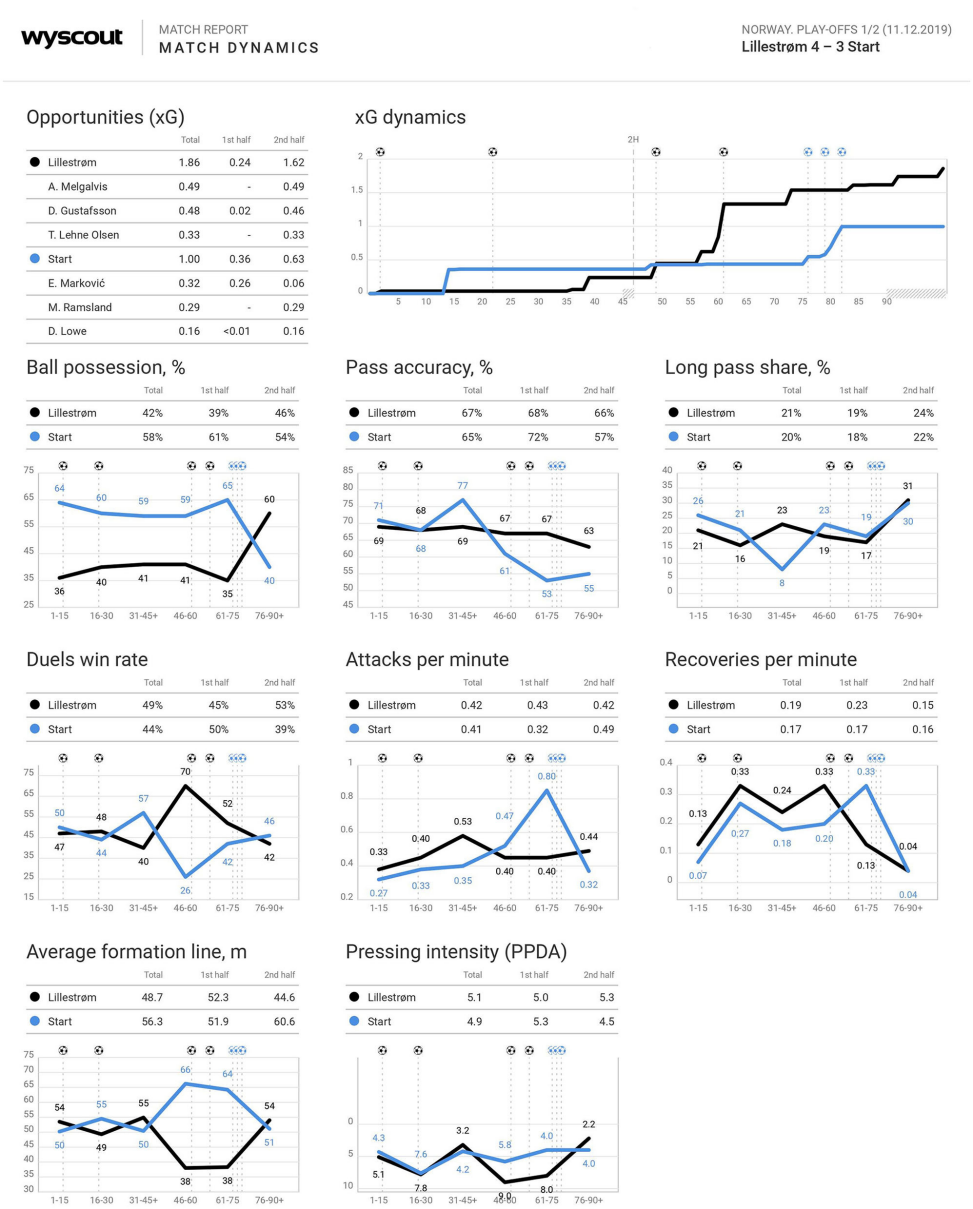
Excerpt of the match report received from Wyscout. Published with permission from Wyscout.

The objective match analyses were received from Wyscout (Figure 2). A closer inspection of the match analyses revealed that IK Start had the highest percentage of *ball possession*, except on the last 15 mins of the game. Regarding *expected goals* (xG), both teams were more effective than anticipated from the expected scoring probability. LSK achieved an xG number of 1.86 and IK Start got a xG of 1.00. IK Start gradually increased their number of *attacks per minute* until the 75th minute where they peaked right before their first goal of the game. Total percentage of *pass accuracy* was quite even for the two teams with LSK achieving a 68% pass accuracy in the first half and 66% in the second half, while IK Start returning 72 and 57% in the first and second halves, respectively. LSK was more successful with regard to *duels win rate* in total compared to IK Start, especially in the second half where LSK achieved 53% compared to 39% for IK Start. More specifically, building up to their first goal, IK Start increased their “duels win rate” from 26 to 42%, while LSK decreased from 70 to 52%. In addition, IK Start increased “attacks per minute” from 0.40 to 0.80, while LSK were stable on 0.40 in the period from the 46th to the 75th minute. Finally, IK Start increased “recoveries per minute” from 0.20 to 0.33, while LSK decreased from 0.33 in the period from 46th to 60th minute to 0.13 in the period from 61st to 75th minute.

**Figure 3:**
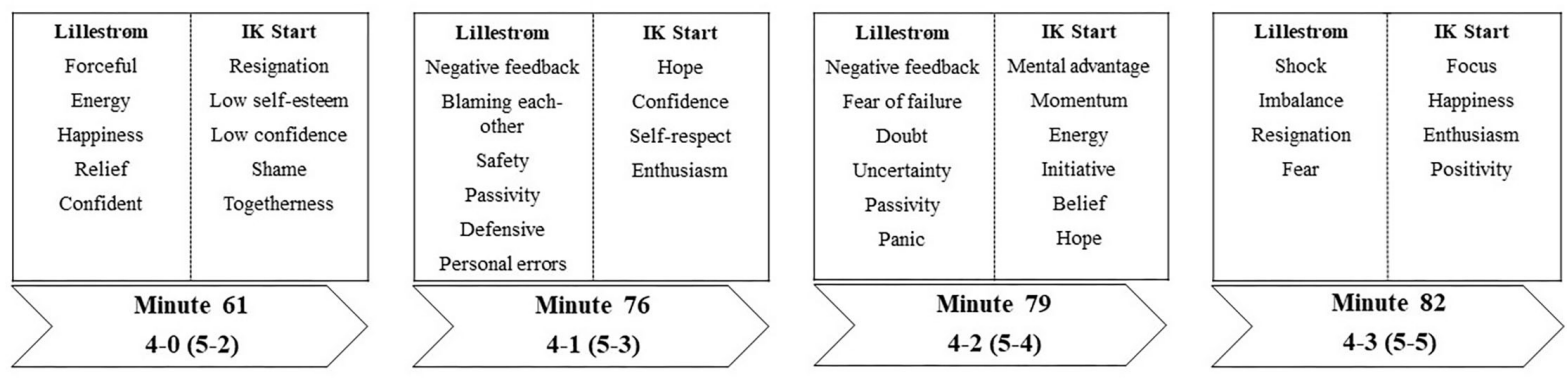
Behavioral, cognitive, and emotional outcomes related to critical match-incidents from 4-0 (agg., 5-2) to 4-3 (agg., 5-5).

The results from the semi-structured interviews are presented in order of importance and critical events for the outcome (see Figure 3). Interview data was organized into four higher order themes relating to the four last goals scored in the game (4-0, 4-1, 4-2, and 4-3). These higher order themes were then outlined in 40 lower order themes divided between the two competing teams. In addition, the results section starts with an introduction with descriptions and general information of what happened before the score was 4-0. This is done to gain a holistic view of how the game had unfolded prior to 4-0.

### Before the Game

As stated earlier, the study aim was to investigate the second game of the two-match qualification between IK Start and LSK. This very important game would decide if IK Start would be promoted to the Eliteserien or whether LSK would retain their place in the Eliteserien. This decisive game was naturally described as very special and crucial by the players:

“*It was a special match for both teams and a lot were at stake*” *(Captain)*

“*The coach was telling us before the game, no matter what happens, positive or negative, we have to keep on going no matter what, because it is only ninety minutes that could separate us from getting to Eliteserien or to not make it” (Goalkeeper)*.

### First Half, 2-0 to LSK, “The First Shock”

In the second minute of the game, LSK scored the first goal after they had regained possession outside the 18-yard box of IK Start and the LSK left-back managed to score a goal. After 22 min, LSK increased their lead when they managed to cross the ball into the penalty area where the LSK striker headed the ball toward the goal and the IK Start center-half attempted a clearance but instead redirected it into the goal.

Even from the beginning of the match, the players recognized that LSK, as a team, were playing with confidence and commitment and that they were on a “higher level” compared to their performance in the first qualification match. LSK had the home advantage in the second game, and with the help of their supporters (who are acknowledged as some of loudest and most passionate supporters in Norway, authors comment), they attacked from the first second of the game. The IK Start players also perceived that the LSK players played with extreme dedication and aggression, which became difficult for IK Start to handle:

“*You notice already from the start, before the goals, that they have an extreme commitment, everybody is running, it seems like they have three players more than us on the pitch, and it's an enormous engagement and push. The home audience was crazy, we almost didn't manage to talk to each other on the pitch, and it seemed like the LSK players managed to play with a higher intensity than us” (Striker)*

“*You feel small. That is maybe the best word I can use to describe it. The energy that they had, and the joy and positivity they exhibited, and the push from the home supporters made you feel small. It felt like we were less than them” (Captain)*.

### Half Time Break, “A Strong Energy”

At half time, LSK were leading 2-0. Nevertheless, match statistics (Figure 2) from the first half showed that IK Start had the highest number of important key performance indicators such as ball possession, pass accuracy, duels win rate, and expected goals (Figure 2). LSK returned better match statistics regarding attacks per minute and recoveries per minute. Even so, all the players agreed that LSK dominated the first half and that the focus for the half time break was how to recover and turn the game around:

“*We were not broken in any ways even though we were down with two goals, it was a strong energy in the dressing room and in the group that this is something we will manage” (Coach)*

“*It was a bit like I thought for myself that LSK has gone the whole autumn without winning football games, they have been leading games before they have gotten a slap in the face, a quite powerful one also. They have been leading a lot and struggled to win these games they are leading. So, I had that in the back of my head the whole time, that they feared to make a mistake, not having the enjoyment of completing something” (Striker)*.

### Second Half, “From Bad to Worse”

As shown in Figure 2, IK Start increased the number of attacks per minute in the first quarter of the second half. Nevertheless, the result went from bad to worse for IK Start. After 61 min, the score was 4-0 to LSK, and everything indicated that LSK would stay in the top division. All respondents highlighted how enormously passionate and emotional the celebration of the LSK players was when they scored the third and fourth goals. They also reflected that the LSK players must have experienced a great relief:

“*They were extremely fired up, and it was an exceptionally energy in them, and it was a force that just hit us like a wave. I noticed when they got 3-0, then I remember one of the LSK players cried out of joy. Then you noticed that they started to feel like the game was going their way” (Captain)*

“*They cried on the pitch, we were down three or four goals, and then you have players on the opposite team that cries. It was probably an enormous relief for them that it was going their way. They have had a huge pressure, maybe a lot bigger than we have had on us, and a club that never has been relegated. So, they probably felt much on that relief” (Sporting director)*.

Their own reflections concerning their own thoughts can be illustrated in the following statements:

“*It's finished now, we have thrown away our opportunity, you just want to be finished and travel back home” (Captain)*

“*I think it's over, hope that the clock will go quickly, get on the bus, get home, take vacation, and come back stronger next season. But we have decided to not give up. With our fans, they create a good atmosphere, and they don't give in, so after we gather ourselves, we agree that we don't give up” (Striker)*.

### Substitution and Tactical Adjustment

In the 71st minute, the coaching staff of IK Start made a double substitution to shift the dynamics of the game. The formation of the team was changed from 1-4-2-3-1 to 1-3-4-1-2 with a central defender as a target man up front, where IK Start adopted a more direct approach compared to earlier in the game.

“*Plan B—It involved that we would move one central defender from defense up as a striker, and yes be more direct in our way of playing. Get the ball as often as possible against their goal, or into dangerous areas around their goal, and that we had players that were ready to chase the long balls that we played” (Coach)*

One of the IK Start players emphasized that this strategic double substitution gave a new mindset to the team:

“*I remember that we got some new guidelines that we were supposed to follow and a different focus, and that felt good since we got stuck in what we tried to do before this change. Now the plan was very clear, it was to play long balls, and just work from there. So, I remember that change, and I recall that it was something that changed our mindset” (Captain)*.

### 4-1, “A Lucky Goal and Increased Confidence”

In the 75th minute, IK Start was awarded a freekick inside the half of LSK and the freekick was crossed into the box resulting in a headed goal. According to the players, there was nothing in the game that would indicate that IK Start would score a goal at that point of the game and they all seemed incredibly surprised:

“*It came out of the blue. Like I said, I felt the game was over. I remember prior the first goal that they had just done an easy mistake and made a freekick out on the side. The ball was kicked into the box, and then we scored. But I still though it would be difficult and that we really just made the end result a bit better” (Captain)*

“*I experienced that it came from a completely harmless position, we just kicked the ball up, and then it is headed into the goal. Then I got some of my self-respect back, it was embarrassing before that*” *(Striker)*

“*Was just a coincidence that came out of the sk*y. *Lucky goal, out of nothing” (Goalkeeper)*.

The players perception of what this goal meant to them was centered around regaining self-respect, confidence, and hope, all positive effects following the tactical change:

“*It was good to get a goal for my confidence going into next season. Not so embarrassing when I scored on the one chance I got. When it's all dark then we don't have anything to lose, so I am very calm, and I will be ready the last twenty minutes. If the ball arrives then I will take care of the chance as best as I can. So, I feel, especially after that goal, that I am very mentally present and focused, even though it may seem like we don't create anything in large parts of the game and that we lose the ball to easy. But I say to myself that if the ball comes, then I will score*” *(Striker)*

“*It was no kind of celebration or things like that. It was more like, yes that happened, now we just keep on going. I don't think it was a strong belief that we would turn the game, but it was nice to score. With the tactical changes we had done, and that the momentum of the game had changed a bit and things like that, it became a positive thing*” *(Captain)*

“*After the first goal there was like hope coming or racing inside of me*” *(Goalkeeper)*.

While this goal increased the belief and the self-respect within the IK Start team, it is important to note that the IK Start players observed the creation of a negative atmosphere among the LSK players that had not been present earlier in the game:

“*I remember that they are shouting and screaming that this is bad and shit, and who did this mistake and trying to blame somebody. From having completely control and almost celebrate the victory and keep their place in the league, it quiet downs a little. The commitment, intensity and energy they had in the first half, it disappears. Now they are just going to safe it” (Striker)*

“*We were all of a sudden very enthusiastic, and they kind of had a feeling of “Oh my god” we could lose something here now. Because until then I think we didn't even have a shot on goal, so we had absolutely no chance, and then like a miracle happens and we scored our first goal out of nowhere and that was, at that day the first negative experience for LSK and the first positive experience for us. Because they got more passive, they didn't push us from that moment on. They, suddenly they became a little defensive and yeah not that aggressive anymore.” (Goalkeeper)*

“*LSK starts doing mistakes they ordinary would not do, or that they had not done through the game. They became, just on that first goal, very affected by it” (Coach)*.

### 4-2, “Game On!”

Just 3 mins after the first IK Start goal, the center back of LSK made a personal error and mistimed a header that gave the striker of IK Start an opportunity alone with the goalkeeper. He managed to exploit this chance and scored the goal to make it 4-2. The perception of the player of their own team indicated that this goal was different from the first goal because it meant something more. Now, they started to believe that they could beat LSK and experienced that the game momentum was suddenly on their side. They gained more energy and recognized the reaction from the supporters, adding yet more belief:

“*Now, I felt that we got the mental advantage of the game even though we still needed one more goal. We had scored two goals on two chances. I sensed that we had control, but still, you think that this is too good to be true, that we should turn this around. So, in retrospective, it's natural for me to say that I felt we had the upper hand in some ways, but I don't know if I really believed it. Because it becomes surreal that we are going to make it even though we were so close and had the momentum. It was special, like, do you dare to believe?*” *(Striker)*

“*The energy that we had been facing from the beginning of the game (from LSK), it was in us now, we had the upper hand*. *Here we actually understand that we have a chance, a quite good chance after how the game has unfolded the last minutes, with the match completely turned around. We had the initiative and the momentum since we got another goal*” *(Captain)*.

Their observation of LSK players were relatively unambiguous. They perceived that a negative atmosphere with less constructive communication and increased shouting, yelling, and complaining had spread in the team. They also highlighted that the LSK players seemed passive, inhibited, and cautious in the way they played. The observations indicated that the negative atmosphere among LSK players was reinforced, and they perceived that the fear of failure was evident in the LSK team:

“*How I saw it, a couple of the players (on LSK) that had that high energy in the start of the game in terms of giving signals to their team members, not that they were the ones that were shouting a lot, but they gave many signals to the players around them, they suddenly became uncertain and passive. And those that had been very verbally active (on LSK) in a positive manner became more verbally active in a negative manner” (Captain)*.

The IK Start captain also describes an uncertainty and passivity among LSK players after the 4-2 goal:

“*We had the advantage, they started to yell at each other, much shouting back and forward, a kind of uncertainty and passivity. You felt in some situations that they instead of taking one step forward for example, they rather awaited and held back a bit to secure the lead that they had. The two central defenders (on LSK) that had been dominant started to lose duels, started to blame others. They were very involved in the first goal at least, and after that they struggled to maintain, what should you call it, a constructive and positive behavior” (Captain)*.

The same was also observed on the sideline by the coach and the sporting director:

“*You get very passive, you become doubtful, and you see that the players start looking around and are unsure on what to do with the ball when they get it. The movements stops and you become… You are too late on the first ball and the second ball, and the uncertainty just grows” (about what he perceived in LSK) (Coach)*

“*Now we see in a way that the LSK players start to… They fear the consequences. While IK Start can just keeping doing what they do” (Sporting director)*.

The second IK Start goal also influenced the audience where the IK Start fans started to believe and therefore created more sound, helping the IK Start players, whereas the LSK fans became more silent. That uncertainty had also spread among the home supporters. The energy the IK Start players acquired from their supporters can be illustrated in the following statement:

“*This is where the hope is ignited. I notice it especially with the IK Start supporters that goes crazy. I think I never have experienced supporters like that before. It was an insane atmosphere, and they were jumping and cheering like crazy on the side with Kevin and Aron (IK Start players). Then I realized, okey, now we start to believe we can do this. This is, now is the possibility to take them, we saw that on the fans and on our players. And it was a bit like, we have not been good, but now we have scored two goals in four minutes*” *(Striker)*.

### 4-3, “The Comeback Is Fulfilled”

In the 82nd minute, IK Start scored the third goal after a long pass from their own half which no LSK defenders attacked. This led to the ball being headed behind the LSK defense, where the IK Start attacker controlled the ball and scored his third goal in just a 6-min period. The game had been completely turned around, and at this moment, IK Start were heading for the premier division in Norway on the away goal rule. The perception of players of their own team was characterized by shock and happiness of what they managed to do, but also the rapid realization that there were still 8 mins plus extra time left, and that the game could still be lost:

“*Like an out-of-body experience. It is like four seconds where you don't understand what has occurred, just a feeling that is impossible to describe. But then it suddenly hits me what is yet to come, and that this is not over. But when we scored, it was huge, huge happiness first*” *(Captain)*

“*We were enthusiastic, and we just did something very special, coming back from 4-0 defeat out of nothing. So, there was extremely positivity, but still a very focused group*” *(Goalkeeper)*.

The IK Start players also described the LSK team to be overflowing with emotions and fear. The collapse permeated throughout the whole LSK team and the perception of the player of the LSK players can be illustrated by the following quotes:

“*They were stunned and just shocked over what had transpired. I think they just needed a little time to realize it. They were not in balance; they were in imbalance and didn't understand what had occurred” (Captain)*

“*It was a surreal feeling. Then you got a sense that they had given up in a way, that they didn't manage to think clearly. They yelled at each other and LSK players were crying on 4-0, but six minutes later it was almost like they cried out of sorrow, it seemed like they had given up then” (Striker)*.

### Final Retrospective Reflections

Idrettsklubben (IK) Start managed to defend the last part of the game, including 10 mins of extra time. The game ended 4-3 to LSK, 5-5 over two legs, meaning that IK Start qualified for the highest division in Norway on the away goal rule. In their reflections on why LSK collapsed, the respondents highlighted “luck” as a catalyst for the start of their comeback:

“*I think luck is a factor, that we got the first goal, without it we would not have made it” (Captain)*

“*That was like all that we talked about before the game and in the half time and also in the circle, that one goal can change the whole game” (Goalkeeper)*.

Furthermore, the goals created a clear change in the way LSK players appeared on the pitch after IK Start managed to score their first goal. In the first 75 min of the game, they had been dominant, aggressive, and in control of the match. However, after the first goal by IK Start, everything changed both collectively and individually for LSK:

“*On 3-0 and 4-0 especially one player on LSK lies down on the pitch and cries and hits down on the grass. He has tears because he is so happy. On 4-1 I get the impression that the whole stadium quiets down a bit, so you notice it on the home supporters. They get some fear even though it is far from being any danger, so you notice it on the audience. Then especially on 4-2 you notice it on the LSK players that it is panic, fear of yet again lose a lead and not winning the qualification. But you notice, maybe before that 4-3 goal that they are a bit out of balance, it is not that intensity, energy, and push that they had in the first half and in the start of the second half. It is kind of gone.” (Striker)*

“*You can say that the positive communication and the “high fives” through the game (in LSK), and look at each other with the winning posture and cheer for each other in all sorts of ways, tackles and duels that they win, it completely disappears” (Coach)*.

In addition, the respondents state that seeing LSK players change their behavior after IK Start scored gave them more belief and energy that they could win the qualification match:

“*After this (the 4-2 goal) then we have that drive and an enormous energy and then you see, I remember them showing some kind of uncertainty and dissatisfaction, and that gave me even more belief that we actually could do this” (Captain)*

“*We scored two goals in three minutes or something like that, and it is still 25 minutes to go, maybe half an hour including extra time. I feel we are being lifted by our supporters, but also from that the home audience is completely quiet, not like anything before in the game. LSK players are yelling at each other and hold their hands in front of their faces. Then I think for myself, we can do this” (Striker)*.

## Discussion

The present study investigated the perceptions and reflections (from the IK Start players, coach, and sporting director) of the radical change in performance in the qualification match between LSK and IK Start. Based on the analysis, the radical change can be described as a collective collapse in the LSK team. According to theory, collective collapse occur more often at the end of “high pressure” matches and where a great deal is at stake (Wergin et al., 2018). It is reasonable to say that the match between LSK and IK Start was a high-pressure game with a great deal at stake. The pressure was probably more extreme for the LSK players representing a club that had not only played the current season at the elite level, but also had a long and successful 45-year club history that never experienced relegation. On the other hand, IK Start was a first division club and are therefore perceived as the underdogs^1^ In addition, IK Start had been the best team in the first match and had a 2-1 lead before the final match. They, therefore, had an advantage in terms of the result and most likely also the psychological benefits of self-confidence and collective efficacy (Chow and Feltz, 2008; Feltz et al., 2008; Van Lier et al., 2015). The match started very well for the LSK team. They experienced a positive flow and had the momentum for the first 60 min by scoring four goals. Suddenly, in a period of 6 min, a momentum shift occurred, and IK Start scored three goals in succession.

The participants reported that in the first 60 min of the match, the emotions and behaviors of the LSK players were highly affected by the goals they scored. For each goal, they perceived increased relief and happiness against the opposing team, and a “We are going to make it” attitude seemed to gradually spread within the team. Several researchers have revealed that athletes report emotions such as happiness, enjoyment, and pride following successful performances (Szabo and Bak, 1999; Wilson and Kerr, 1999; Ruiz and Hanin, 2004; Cerin and Barnett, 2006). Furthermore, expressing positive emotion (e.g., happiness, enjoyment, pride) has been suggested to promote and boost confidence and to signal dominance and superiority (Brown and Marshall, 2001; Tracy and Robins, 2007a,b; Tracy and Matsumoto, 2008). The emotional expression may also be reinforced by an emotional contagion process in the team. More specifically, this phenomenon claims that expression of the emotions experienced by an individual can transfer to other individuals nearby, particularly when one holds a close relationship with them (Hatfield et al., 1994; Kelly and Barsade, 2001). In an achievement setting, Barsade (2002) found that positive emotion contagion improved cooperation, decreased conflict, and increased perceptions of task performance. In contrast, contagion of negative emotions led to the reverse. It is reasonable to suggest that during the first 60 min, positive emotional (LSK team) and negative emotional (IK Start team) contagion was present. However, even if all respondents expressed that despair, shame, and an acceptance of defeat were soon-to-be a reality, there was still an attitude in the team that they had to fight to the end of the match, at least to prevent an even more humiliating result and to maintain their self-respect. This “fight to the end” attitude at the individual level demonstrates the attributes of mental toughness which is reported in elite athletes (Thelwell et al., 2005; Danielsen et al., 2017). At the team level, it may be a characteristic of a resilient sports team. Morgan et al. (2013) defined team resilience as “a dynamic psychosocial process which protects a group of individuals from the potential negative effect of the stressors they collectively encounter. It comprises of processes whereby team members use their individual and combined resources to positively adapt when experiencing adversity” (p. 552).

The match statistics (Figure 2) indicate that something was changing in the game from the 46th to 60th minute to the 61st to 75th minute regarding KPI variables (e.g., duels win rate, attacks per minute, recoveries per minute). In sum, the KPI variables may indicate that game dynamics were shifting. However, none of the respondents reported to perceive those changes during that period of the game. According to the theory of collective collapse, specific triggers or critical events are usually reported as prevalent. These triggers or critical events start the accumulation process toward the actual collapse (Apitzsch, 2009; Wergin et al., 2018). Although it is difficult to exactly pinpoint what is the specific starting point that caused the collapse, the study has identified some factors and critical events that may shed light on the process. Nevertheless, it is likely that they are all interrelated and mutually reinforced in the accumulation process. Firstly, the emotional and behavioral expression (happiness, relief, overconfidence) of the LSK players as a reaction to their goals may subsequently have contributed to a small gradual reduction in their effort and performance and, as such, “invited” IK Start into the game. Based on the responses of respondents, there is no evidence for such an explanation, but the effect can have been mostly psychological. The invitation may have caused, for example, a small increment in efficacy by the overconfidence expressed by LSK (Wergin et al., 2018). Moreover, the positively expressed emotions in the LSK team and their own feeling of shame and hopelessness in IK Start could have contributed to an increased “stand together” feeling and strengthened the team cohesion (Høigaard, 2020). For example, Turner et al. (1984) demonstrated in a laboratory setting that negative experiences, like failure or defeat, may contribute to increase group cohesion, particularly if the commitment and team identification is high. Secondly, the tactical shift and substitution after 71 min could have created an imbalance in the match that LSK were unable to re-address. This may have been due to their overconfident attitude (e.g., we are going to win anyway). The perception of the player was that the reorganization and the updated instructions were significant, albeit psychological, within the IK Start team. There is some evidence that in situations perceived as chaotic and unsure or when the level of anxiety is high, an attempt to break the negative pattern through basic task and structure initiate hope, increase the feeling of control, and, subsequently, change of effort and attitude (Jones and Harwood, 2008; Apitzsch, 2019). Finally, the most important trigger according to the respondents was the first IK Start goal. Even if the goal was explained as a “gift from heaven” and the players perceived it as coming from nowhere, it was a game changer. According to the respondents, the goal triggered a set of positive cognitive, emotional, and behavioral reaction chains (e.g., positive momentum) in IK Start, which was further increased in line with the next match goals. In contrast, they perceived a negative reaction chain in the opposing team. The perception and interpretation of players of the three IK Start goals and how the two teams responded indicated two different behavioral and emotional trajectories. The reaction of IK Start players to their own goals was centered around positive emotions and thoughts (e.g., hope, belief, happiness) that generated productive behavioral outcomes. On the other hand, the IK Start players perceived negative emotions and counterproductive behavior among the LSK players (e.g., passivity, panic, negative feedback). According to these findings, one may argue that there was a positive intra-team contagion in the IK Start team. In addition, an inter-team contagion seemed to have been evident based on the perception of the opposing team (i.e., the perception of the negativity in LSK additionally increased the positive momentum in IK Start; Jones and Harwood, 2008). According to Taylor and Demick (1994), such inter-team emotional contagion may have a contrasting effect and, therefore, could reinforce the emotions that were prevalent in both teams.

The reactions of IK Start players to the first IK Start goal was that it was a motivational boost that increased their belief that they could challenge LSK. This belief, in addition to celebration, happiness, and pride, considerably increased when they scored their two next two goals. Further, the positive emotions seemed to be transferred between the players and additionally reinforced the emotion and thoughts in line with the proposal of the emotional contagion theory (Barsade, 2002). This is relevant as former research has linked positive emotion to several positive performance-related outcomes, such as increased confidence, courage, attentional focus, reduced fear of failure, and increased effort (Izard, 1991; Moll et al., 2010). In contrast, a reverse mechanism was prevalent in the LSK team. The players perceived changes in the body language and gesticulatory and facial expressions of LSK players after the 4-1 goal, which they interpreted as small signs of shock, amazement, astonishment, and insecurity. The next goal accelerated this negative body language, and the respondents also reported that they perceived that more negative communication occurred (i.e., yelling, blaming) in addition to less coordinated play. Within LSK, there appeared to be hectic play within a disorganized unit which lead to a breakdown in coordination, often associated with disjointed shared mental models (Reimer et al., 2006; Eccles, 2010). When team coordination deteriorates and negative communication spreads through emotional contagion, the influence of athlete leaders has been reported to be of great importance (Cotterill et al., 2020). Former research has, for example, identified athlete leaders as vital for several different team functioning factors (Crozier et al., 2013; Loughead et al., 2014; Cotterill and Fransen, 2016).

Finally, during the end of the match, it was clear that the behavior and emotional expression the respondents perceived were in line with research that has been identified as factors in a collective collapse process (e.g., anxiety, fear of failure, reduce attentional focus, hectic play, limited and/or negative communication, and decreased performance; Apitzsch, 2009; Wergin et al., 2019). It is reasonable to believe that in the collective collapse process, emotional contagion was prevalent in both teams but with different content and context. However, negative emotions have been found to be spread more rapidly than positive emotions and thus, compound on field issues in the team. There is also evidence that less frequent and tense negative emotion develops more easily than positive emotions (Baumeister et al., 2001; Felps et al., 2006). Parallel to the intra-team social contagion, the process in each team may have also been reinforced by contrasting emotional contagion. Following this reasoning, the goals, the celebrating, and the positive emotion in one team may be further reinforced by shock, disappointment, and negative emotion in the other team and vice versa. The contrasting emotional contagion has been identified in a study of male soccer players' post-penalty emotional expressions by Moll et al. (2010), where they stated that emotional contagion does not only occur between teammates but can also occur between opponents.

The present study has gained an insight into how critical events may contribute to the development of a collective collapse in elite football. The study shows that the critical events (i.e., the IK Start goals) directly contributed to more offensive and positive emotions, thoughts, and behavior among the IK Start players, while respondents observed that the LSK players reacted with insecurity, passivity, and panic behavior. Furthermore, the individual reactions of players seemed to affect the whole team through an intra-team emotional contagion process. In addition, the findings also indicated an inter-team emotional contagion process where emotions and behavior in the LSK team were transmitted to the IK Start team, but with a contrasting effect. Specifically, when the emotional and behavioral expressions (verbal and nonverbal) of LSK players were perceived as despair, panic, insecurity, low self-confidence, and negative communication, the IK Start players further gained their self-confidence, enthusiasm, and engaged in more positive and constructive communication. In summary, this soccer case study highlights the importance and relevance of understanding and investigating collective collapses in sport. Furthermore, the general structure of the process model by Wergin et al. (2019) was identifiable within the data collected in the present study. Nevertheless, the perception and interpretation of the participants on the antecedents, critical events, and subsequent outcomes highlight the complexity of the dynamic processes involved in a collective collapse. Importantly, both intra-team and inter-team social contagion may be prevalent, and thus possibly act as catalysts in the process leading to a collective collapse.

There are some limitations in the current study that must be considered. Firstly, a small sample size (*n* = 5) was utilized in the data collection. However, participants were central figures (i.e., athlete leaders and formal leaders) in both planning and participating in the investigated game which provided enriched information of the phenomenon of collective collapse from different perspectives. Moreover, the study only includes participants from one of the competing teams in the investigated game. The lack of information of how LSK players and coaches experienced the game raises the question of how accurate were the IK Start player perceptions of the emotions of the LSK players. However, it is understandable that the team experiencing the collective collapse, and consequently being relegated, were reluctant to talk about what had transpired and rather wanted to focus on the next season. Additionally, one potential limitation was the possibility to identify the respondents through their specific quotes. This may, in some situations, affect how respondents would answer certain questions. However, none of the respondents raised any counter perceptions regarding anonymity since they were asked to talk about a positive experience that ended in promotion and what some of them described as the greatest achievement in their careers. The possibility of a “glow” effect when recalling former performances is described by Cornelius et al. (1997) and must be taken into account when interpreting these results. Since the investigated game started badly for the IK Start players but ended with a positive result after a clear change of momentum around the 75th minute, all respondents managed to recall and describe both negative and positive periods of the game without being subject to response bias. In addition, even if the objective match statistics from Wyscout provides useful information, it does not provide insight into physical efforts performed by the two teams and by individual players. This raises the question whether physical fatigue was a factor influencing the end result. These KPI limitations should be considered when interpreting the study findings. Finally, the study was neither able to identify nor objectively measure the impact of the emotional contagion based on the study design. This is something future research should seek to explore. However, in elite sport, even small signs or changes (objectively and/or psychologically) may have a significant impact on team performance and results (Boss and Kleinert, 2015; Bourbousson et al., 2015), and it is reasonable to believe that emotional contagion contributes to the collective collapse.

Some practical implications are useful to mention as they can help either to avoid collective collapse or reduce the impact it could have on team performance. Firstly, luck and unexpected changes occur in all sport. For example, the opponent may score even if they do not deserve to despite it being against the “run of play.” Critical incidents that are difficult to handle will happen, therefore, it is important to be prepared and to never give up because the momentum shift can rapidly change again. Secondly, do not show despair or overtly express negative emotions. Body language is interpreted by the opposition and can be exploited, providing hope, motivation, and a sense of belief. In these circumstances, cultural architects (Danielsen et al., 2019) could be useful to counteract collective collapse, negative body language, and negative communication processes. Lastly, coaches need to facilitate team processes, like shared mental models, that can prevent collective collapse and execute strong leadership when it is needed in time of crisis. Future research is encouraged to examine our developed model of how collective collapse is perceived from a bystander perspective to provide guidance to teams, coaches, and sport psychologists in dealing with collective sport team collapse. The investigation of collective collapse in future research should include the perspective from both teams, providing an enriched insight to how the two competing teams experience collective collapse. It would be fruitful to investigate collective collapse in relation to different group dynamic aspects, such as social identity, team resilience, athlete leadership, and cultural architects.

## Data Availability Statement

The raw data supporting the conclusions of this article will be made available by the authors, upon request.

## Ethics Statement

The studies involving human participants were reviewed and approved by Norwegian Social Sciences Data Service. The participants provided their written informed consent to participate in this study.

## Author Contributions

GS, TH, GJ, and RH: conceptualized and designed the study. GS, TH, and RH: assisted in the planning and acquisition of data. GS, TH, GJ, SS, and RH helped with the analysis and interpretation of the data, critically revising the manuscript, and added important intellectual content. All authors gave approval for the final version of this manuscript to be published and agree to be accountable for all aspects of the work.

## Conflict of Interest

The authors declare that the research was conducted in the absence of any commercial or financial relationships that could be construed as a potential conflict of interest.

## Publisher's Note

All claims expressed in this article are solely those of the authors and do not necessarily represent those of their affiliated organizations, or those of the publisher, the editors and the reviewers. Any product that may be evaluated in this article, or claim that may be made by its manufacturer, is not guaranteed or endorsed by the publisher.

## Footnotes

^1^ https://www.tv2.no/a/11034145/
